# Perinatal depression screening and diagnosis: Identifying opportunities to improve optimal care

**DOI:** 10.1007/s00737-026-01676-4

**Published:** 2026-02-06

**Authors:** Kelli Ryckman, Maiti Peters, Erik Parker, Lilian Golzarri-Arroyo, DeShauna Jones, Morolake Adeagbo, Jaime Hamil, Beth Shelton, Hyunkeun Cho, Karen Tabb, S Darius Tandon, Elissa Faro

**Affiliations:** 1 Indiana University Bloomington, Bloomington, United States; 2 University of Iowa, Iowa City, United States; 3 Northwestern University, Evanston, United States; 4 University of Illinois Urbana-Champaign, Urbana, United States; 5 University of California, San Diego, San Diego, United States; 6 Center for Discovery and Innovation, Nutley, United States

**Keywords:** Perinatal depression, Intersectionality, Disparities, Screening, Diagnosis

## Abstract

**Purpose:**

Perinatal depression (PND), occurring during pregnancy or within the first year after childbirth, is a common medical complication with serious consequences when left untreated, including hospitalizations, increased morbidity, and suicide. This study examined the intersection among racial, ethnic, and sociodemographic disparities in PND screening and diagnosis within a Midwestern health system.

**Methods:**

We performed a multilevel analysis of individual heterogeneity and discriminatory accuracy (MAIHDA) using electronic health record data for 79,992 deliveries. The study included women aged 15–50 who delivered live-born infants between 22 and 44 weeks of gestation. PND screening was identified by the presence of a PHQ-9 or EPDS score, and ICD9/10 codes were used to define depression diagnosis within one year before or after delivery.

**Results:**

Across groups older women (≥ 36 years) were less likely to be screened for PND (OR = 0.65; 95%CI = 0.56–0.75) but not less likely to be diagnosed. Non-Hispanic Black, Hispanic, and Asian women were more likely to receive PND screening (ORs = 1.23–1.31) but less likely to be diagnosed (ORs = 0.16–0.60) compared to Non-Hispanic White women. Enrollment in public insurance was not associated with PND screening but was linked to a higher likelihood of diagnosis (OR = 1.41, CI = 1.24–1.61). Women in rural areas were less likely to be screened for PND (OR = 0.66, CI = 0.58–0.75), with no significant association with diagnosis.

**Conclusions:**

Significant disparities exist in PND screening and diagnosis. Groups facing historic structural inequities are more likely to be screened but less likely to receive a diagnosis, highlighting the need for targeted interventions to address these inequities.

## Introduction

Perinatal depression (PND), occurring during pregnancy or within the first year after childbirth, represents a major public health concern with profound implications for maternal and child health. Globally, perinatal depression has been increasing since the COVID-19 pandemic with meta-analyses estimating the prevalences at ~ 21% antenatally and ~ 25% postpartum (Sahebi et al. 2024; Yin et al. 2023). PND often goes undetected and untreated, which can lead to severe maternal health consequences, such as insomnia, poor quality of life, suicidal ideation, substance abuse, hospitalization, morbidity, increased risk of child maltreatment, and impaired mother bonding with the infant (Brown et al. 2023; Fasial-Cury et al., 2021; Kingston et al. 2012; Lusskin et al. 2007). Children exposed to maternal depression in utero or early infancy are at increased risk of prematurity, developmental delays, and long-term emotional and behavioral challenges (Kingston et al. 2012; Lusskin et al. 2007; Simonovich et al. 2021; Tabb et al. 2023). The broader family, including partners, may also experience deteriorating mental health (Dagher et al. 2021). Perinatal depression is a highly treatable condition that can have serious consequences when undetected and/or untreated.

Though screening tools and assessment guidelines are well-established, gaps in practice, disparities in care, and systemic barriers limit their impact. Notably, younger women and those identifying as multiracial or Native American face higher rates of depression during and after pregnancy (Dagher et al. 2021; Daw et al. 2025; Sidebottom et al. 2023). Two studies examining a single health system in Minnesota found disparities in screening and diagnosis persist across racial and ethnic groups (Sidebottom et al. 2021, 2023). For example, Black, Asian, and Native American women were equally likely to be screened as their non-Hispanic White counterparts for prenatal depression, but were less likely to be screened for postpartum depression (Sidebottom et al. 2021). Furthermore, these studies found that despite 11–13% of women screening positive for depression, Black women with elevated screening scores during pregnancy were half as likely as White women to receive a formal diagnosis (adjusted OR = 0.40, *p* = 0.002), a disparity not observed during the postpartum period (Sidebottom et al. 2023). This is important as it is well known that Black, Asian, and multiracial women experience higher rates of depressive symptoms, yet are less likely to receive treatment (Kozhimannil et al. 2011). Structural racism, bias in clinical encounters, and lack of person-centered care contribute to these inequities (Haight et al. 2024; Sidebottom et al. 2023).

Due to the substantial gaps in screening and diagnosis, there remains a strong need for more inclusive research on disparities in PND. Current practices fall short of guideline standards, especially during pregnancy and among marginalized populations (Dagher et al. 2021; Yang et al. 2024). However, there is limited research that holistically examines the intersection of racial, ethnic, socio-economic, and geographic disparities in PND screening and diagnosis. To inform implementation research aimed at equitably integrating screening and diagnosis into routine care, we must first understand the intersection of disparities affecting PND screening and diagnosis. The following study examined the intersection among racial, ethnic, and sociodemographic disparities in PND screening and diagnosis within a Midwestern health system.

## Materials and methods

### Study population

The data for this study were derived from all available, linked infant birth and maternal delivery records dated from January 2013 to March 2024 as obtained from the Indiana University (IU) Health Electronic Health Record (EHR). Access to the IU Health Enterprise Data Warehouse was provided by Regenstrief Institute, Inc. The warehouse includes data from 16 IU health hospitals and clinics. The dataset provided by Regenstrief Institute, Inc. included detailed information on maternal and infant demographics, encounters, and procedure records from at least one year before birth up to one year of age. Infant gestational age, as determined by date of last period or ultrasound, was used to restrict the analysis dataset to include women between 15 and 50 years of age who delivered a liveborn newborn between 22 and 44 weeks of gestation. The data included diagnosis codes based on the 9th and 10th revisions of the *International Classification of Diseases* (ICD). This research study was conducted retrospectively from data obtained for clinical purposes. Approval for a waiver of consent was granted by the Institutional Review Board at Indiana University (22798). Clinical trial number: not applicable.

The primary outcomes of this study were PND screening and diagnosis. PND screening was defined as at least one recorded score of either the Patient Health Questionnaire (PHQ-9) or the Edinburgh Postnatal Depression Scale (EPDS) depression screener during 1 year before or 1 year after childbirth. The PHQ-9 is a self-administered screening tool that assesses the presence of symptoms associated with a major depressive disorder diagnosis (Palmer et al. 2024). The EPDS screens for clinically relevant signs of major and moderate depressive disorder, as well as suicidal ideation, a common symptom of PND (Stefana et al. 2024). PND diagnosis was defined as women having an ICD 9 or 10 code-based diagnosis of depression in any encounter during the 1 year before and 1 year following childbirth. Codes used to define depression were taken from the *Chronic Condition Data Warehouse* definitions of depressive disorders, and included the codes 296.20, 296.21, 296.22, 296.23, 296.24, 296.25, 296.26, 296.30, 296.31, 296.32, 296.33, 296.34, 296.35, 296.36, 300.4, 311, F32.0, F32.1, F32.2, F32.3, F32.4, F32.5, F32.89, F32.9, F32.A, F33.0, F33.1, F33.2, F33.3, F33.40, F33.41, F33.42, F33.8, F33.9, and F34.1.

The variables considered for this analysis, maternal race and ethnicity, age at delivery, insurance status, primary language, and Rural-Urban Commuting Area (RUCA) codes, were all derived from the EHR. Zip codes of maternal residence at time of birth were used to assign RUCA classifications for each individual using information for commuting flows and population size cutoffs of 50,000 + for “Urban”, 10,000–49,999 for “Large Rural City/Town”, and 2,500–9,999 for “Small Rural Town”.

### Statistical analyses

Demographic characteristics are described by number and percent or as mean (standard deviation) and median (interquartile range). Additionally, women were categorized based on screening and diagnosis across three time frames: (1) prenatal: from one year before delivery up to the delivery date, (2) at delivery: from the delivery date through one week postpartum, and (3) postpartum: from one week to one year after delivery. Women could appear in more than one time frame if they were screened or received a diagnosis code multiple times; however, within a single time frame, each woman was counted only once, regardless of the number of screenings or diagnoses.

The overall outcomes used in the intersectionality analysis were a binary variable that included any screening or any diagnosis during the timeframe from one year prior to delivery through one year postpartum. We examined the impact of the intersection of sociodemographic variables on the rates of PND using intersectional multilevel analysis of individual heterogeneity and discriminatory accuracy (I-MAIHDA) models (Evans et al. 2024). This approach sorts all subjects into subgroups (or strata) based on their unique combinations of sociodemographic variables. Then it uses that strata variable as a random intercept in a generalized linear mixed model framework. For each model a logistic mixed model (logit link) was constructed with fixed effects for maternal age, race/ethnicity, insurance status, language, and RUCA category. Each model included a random intercept for social strata, a constructed variable with stratum defined by an individual’s combination of age, race/ethnicity, insurance status, and RUCA category variables. P-values were based on type 3 sum of squares, with a significance threshold of *P* < 0.05. All statistical analyses were performed in R (version 4.3.0) using the *lme4* (version 1.1–35.1; (Bates et al., 2015), and *gtsummary* (version 1.7.2; Sjoberg et al., 2021) packages.

## Results

**Fig. 1:**
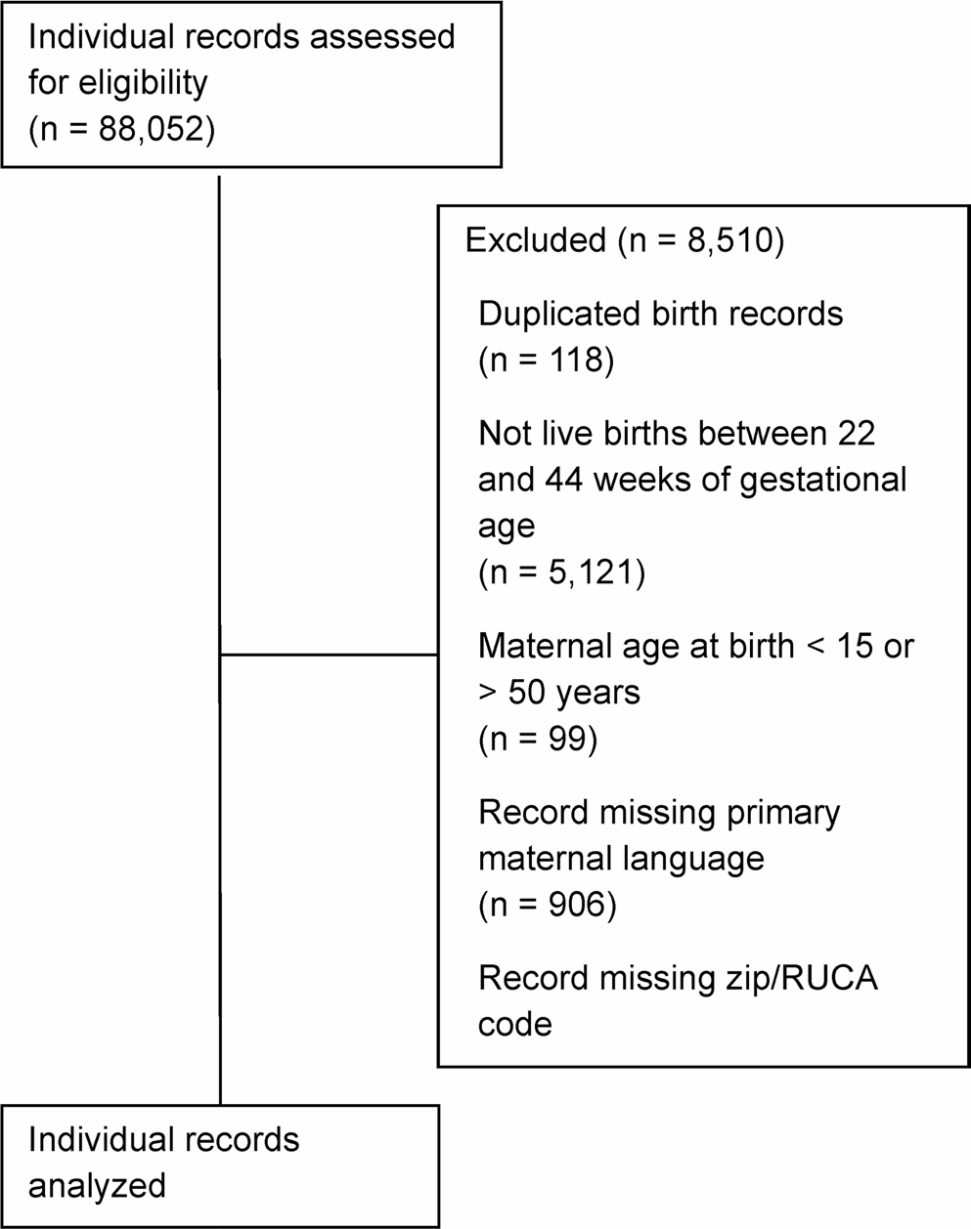
Record inclusion diagram

A total of 79,992 women were included in this study, after excluding 8,510 (9.6%) women who did not meet the inclusion criteria (Fig. 1). Most women included in the study were White non-Hispanic (69%), between 21 and 30 years old at delivery (55%), covered by private insurance (56%), resided in urban-categorized RUCA areas (88%), and spoke primarily English (95%; Table 1). Overall, 90.2% of women were screened for depression sometime during the perinatal period (one year prior to delivery through one year postpartum). Examining rates by time-period 17% were screened during the prenatal period, 88% were screened around the time of delivery, and 17% were screened postpartum. On average, women were screened ~ 3 times during the perinatal period (Table 1). Overall, 14% of women received a diagnosis for depression sometime during the perinatal period, with 6.6% being diagnosed in the prenatal period, 7.3% diagnosed around delivery, and 5.6% diagnosed postpartum (Table 1).

**Table 1:** Demographic characteristics of the study population

Characteristic	*N* = 79,992^*1*^
Maternal Race/Ethnicity	
White non-Hispanic	54,849 (69%)
Black non-Hispanic	12,345 (15%)
Hispanic or Latinx	7,114 (8.9%)
Asian	3,822 (4.8%)
American Indian/Alaskan Native/Native Hawaiian/Pacific Islander	359 (0.4%)
Other/Unknown/Declined	1,503 (1.9%)
Maternal age category	
15–20 years old	8,826 (11%)
21–30 years old	44,144 (55%)
31–35 years old	18,697 (23%)
36 + years old	8,325 (10%)
Maternal insurance status	
Private	44,554 (56%)
Public	34,236 (43%)
No insurance	1,202 (1.5%)
RUCA category	
Urban	70,696 (88%)
Large Rural City/Town	3,455 (4.3%)
Small Rural Town	5,841 (7.3%)
Maternal primary language	
English	75,900 (95%)
Non-English	4,092 (5.1%)
Screened for depression - Overall	
No	7,838 (9.8%)
Yes	72,154 (90%)
Screened for depression - Prenatal	
No	66,675 (83%)
Yes	13,317 (17%)
Screened for depression - Delivery	
No	9,350 (12%)
Yes	70,642 (88%)
Screened for depression - Postnatal	
No	66,097 (83%)
Yes	13,895 (17%)
Number of depression screenings - Overall	
Mean (SD)	3.53 (3.99)
Median (Q1, Q3)	2.00 (2.00, 4.00)
Depression diagnosis - Overall	
No	68,890 (86%)
Yes	11,102 (14%)
Depression diagnosis - Prenatal	
No	74,710 (93%)
Yes	5,282 (6.6%)
Depression diagnosis - Delivery	
No	74,174 (93%)
Yes	5,818 (7.3%)
Depression diagnosis - Postnatal	
No	75,535 (94%)
Yes	4,457 (5.6%)

^*1*^ n (%)

Among women who had a depression diagnosis prenatally, 23.7% were screened during the prenatal period, 50.6% were screened around time of delivery, and 20.9% were screened postnatally (Table 2). Among those who had a depression diagnosis around the time of delivery, 19.7% were screened prenatally, 56.2% were screened around the time of delivery, and 19.5% were screened postpartum. Among women who had a depression diagnosis postnatally, 19.2% were screened prenatally, 49.9% were screened at the time of delivery, and 27.0% were screened postpartum. Among women without a diagnosis of depression, 11.3% were screened prenatally, 69.2% were screened around the time of delivery, and 11.6% were screened postpartum (Table 2).

**Table 2:** Screening and diagnosis rates by perinatal time-points

		Diagnosis			
	Diagnosis	Prenatal	Delivery	Postnatal	No Diagnosis
Screening	Prenatal	2,165 (23.7%)	1,816 (19.7%)	1,511 (19.2%)	9,904 (11.3%)
	Delivery	4,619 (50.6%)	5,187 (56.2%)	3,933 (49.9%)	60,829 (69.2%)
	Postnatal	1,907 (20.9%)	1,800 (19.5%)	2,130 (27.0%)	10,147 (11.6%)
	No Screening	433 (4.7%)	427 (4.6%)	315 (4.0%)	6,966 (7.9%)
		9,124 (100%)	9,230 (100%)	7,889 (100%)	87,846 (100%)

Women were categorized based on screening and diagnosis across three time frames: (1) prenatal: from one year before delivery up to the delivery date, (2) at delivery: from the delivery date through one week postpartum, and (3) postpartum: from one week to one year after delivery. Women could appear in more than one time frame if they were screened or received a diagnosis code multiple times; however, within a single time frame, each woman was counted only once, regardless of the number of screenings or diagnoses

**Table 3:** MAIHDA strata with the 5 highest and lowest predicted incidences of **(A)** *administration* of the Edinburgh postnatal depression scale (EPDS) and/or patient health questionnaire (PHQ-9); and **(B)** perinatal depression as diagnosed from ICD 9/10 codes from the MAIHDA models

	A) PND screening				B) PND diagnosis			
	Rank	Strata	Strata (*N*)	Predicted % (95% CI)	Rank	Strata	Strata (*N*)	Predicted % (95% CI)
Bottom 5 lowest strata								
	**1**	36 + years old, Other/Unknown/Declined race, No insurance, Small Rural Town	< 10	75.19 (67.36–81.05)	**1**	36 + years old, Asian, No insurance, Urban	10	1.25 (0.73–2.06)
	**2**	31–35 years old, Other/Unknown/Declined race, No insurance, Small Rural Town	< 10	76.72 (69.65–82.85)	**2**	21–30 years old, Asian, No insurance, Urban	29	1.32 (0.77–2.11)
	**3**	36 + years old, White non-Hispanic, No insurance, Small Rural Town	16	77.80 (71.40–82.65)	**3**	31–35 years old, Asian, No insurance, Urban	24	1.34 (0.80–2.20)
	**4**	31–35 years old, White non-Hispanic, No insurance, Small Rural Town	35	78.91 (73.40–83.51)	**4**	15–20 years old, Asian, No insurance, Urban	< 10	1.88 (1.12–3.23)
	**5**	21–30 years old, Other/Unknown/Declined race, No insurance, Small Rural Town	< 10	78.97 (72.22–84.17)	**5**	31–35 years old-Hispanic or Latinx, No insurance, Small Rural Town	< 10	2.03 (1.19–3.37)
Top 5 Highest strata								
	**166**	15–20 years old, Hispanic or Latinx, Public insurance, Urban	907	94.42 (93.00–95.57)	**166**	36 + years old, White non-Hispanic, Public insurance, Small Rural Town	154	23.28 (18.16–30.04)
	**167**	21–30 years old, American Indian/Alaskan Native/Native Hawaiian/Pacific Islander, Private insurance, Urban	119	94.63 (91.28–96.62)	**167**	15–20 years old, White non-Hispanic, Public insurance, Large Rural City/Town	179	23.80 (18.68–29.95)
	**168**	21–30 years old, American Indian/Alaskan Native/Native Hawaiian/Pacific Islander, Public insurance, Urban	65	94.69 (91.55–96.68)	**168**	21–30 years old, White non-Hispanic, Public insurance, Large Rural City/Town	721	24.31 (20.46–29.20)
	**169**	15–20 years old, American Indian/Alaskan Native/Native Hawaiian/Pacific Islander, Public insurance, Urban	10	95.43 (92.41–97.18)	**169**	31–35 years old, White non-Hispanic, Public insurance, Urban	2189	24.46 (21.03–28.51)
	**170**	15–20 years old, American Indian/Alaskan Native/Native Hawaiian/Pacific Islander, Private insurance, Urban	< 10	95.59 (92.58–97.22)	**170**	31–35 years old, White non-Hispanic, Public insurance, Large Rural City/Town	188	25.56 (20.02–31.87)

^1^ OR = Odds Ratio, CI = Confidence Interval

The age, race/ethnicity, insurance, and RUCA variables together created 170 unique strata. Of these strata, the White non-Hispanic − 21–30 years old - private insurance- urban strata was the largest with 15,986 (19.98%) women. The average stratum size across all strata was 471 women (standard deviation of 1,676), while the median size was 20.5 women (25th quantile: 3.25, 75th quantile: 185.75 mothers) per stratum.

First, we identified differences by sociodemographic characteristics in PND screening. Women in the youngest age group (15–20 years old) at delivery had significantly higher odds of being screened using PHQ or EPDS compared to all other age groups (*p* < 0.001; Table 4A). All racial/ethnic groups (except for the Other/Unknown/Declined category) had significantly higher odds of PND screening as compared to White non-Hispanic women (*p* < 0.001) with Black non-Hispanic, Hispanic or Latinx, Asian, and American Indian/Alaskan Native/Native Hawaiian/Pacific Islander women showing increased odds of screening of 23%, 39%, 31%, and 96%, respectively. Women with no insurance were 26% less likely to be screened compared to mothers with private insurance (odds ratio [OR]: 0.74, 95% confidence interval [CI]: 0.61–0.90, *p* = 0.008). Women living outside of urban areas were less likely to be screened, with those living in large rural cities or towns being 20% less likely (OR: 0.80, CI: 0.68–0.93, *p* < 0.001), and those in small rural towns being 34% less likely (OR: 0.66, CI: 0.58–0.75, *p* < 0.001) as compared to women living in urban areas. Finally, primarily non-English speaking women showed 31% higher odds of being screened (OR: 1.31, CI: 1.13–1.51, *p* < 0.001) as compared to primarily English-speaking women. The five strata with lowest predicted prevalence of PND screening (75–79%) were comprised mostly of Non-Hispanic White women, or those who did not identify their race, who had no insurance, were generally older and living in small rural towns, conversely the five strata with highest prevalence of screening (94.4–95.6%) were mostly younger, Hispanic, American Indian, Alaskan Native, Native Hawaiian or Pacific Islander with private or public insurance who live in urban areas (Table 3A).

**Table 4:** MAIHDA model results for three outcomes

	A) PND screening			B) PND diagnosis		
	OR^1^	95% CI^1^	*p*-value	OR^1^	95% CI^1^	*p*-value
Maternal age category			**< 0.001**			0.118
15–20 years old	—	—		—	—	
21–30 years old	0.82	0.72, 0.93		0.84	0.70, 0.99	
31–35 years old	0.71	0.62, 0.82		0.84	0.70, 1.01	
36 + years old	0.65	0.56, 0.75		0.94	0.78, 1.15	
Maternal Race/Ethnicity			**< 0.001**			**< 0.001**
White non-Hispanic	—	—		—	—	
Black non-Hispanic	1.23	1.08, 1.41		0.6	0.50, 0.72	
Hispanic or Latinx	1.39	1.20, 1.61		0.51	0.42, 0.61	
Asian	1.31	1.10, 1.56		0.16	0.12, 0.21	
American Indian/Alaskan Native/Native Hawaiian/Pacific Islander	1.96	1.24, 3.10		0.47	0.30, 0.72	
Other/Unknown/Declined	0.88	0.72, 1.06		0.39	0.30, 0.51	
Maternal insurance status			**0.008**			**< 0.001**
Private	—	—		—	—	
Public	0.94	0.86, 1.04		1.41	1.24, 1.61	
No insurance	0.74	0.61, 0.90		0.62	0.48, 0.81	
RUCA category			**< 0.001**			0.212
Urban	—	—		—	—	
Large Rural City/Town	0.8	0.68, 0.93		1.18	0.98, 1.42	
Small Rural Town	0.66	0.58, 0.75		1.07	0.90, 1.28	
Maternal primary language			**< 0.001**			**< 0.001**
English	—	—		—	—	
Non-English	1.31	1.13, 1.51		0.36	0.30, 0.44	

In model **A** the outcome was *administration* of the Edinburgh postnatal depression scale (EPDS) and/or patient health questionnaire (PHQ); and for model **B** the outcome was perinatal depression as diagnosed from ICD 9/10 codes alone. For each model a logistic mixed model (logit link) was constructed with fixed effects for maternal age, race/ethnicity, insurance status, language, and Rural-Urban commuting area code (RUCA) category. Each model further included a random intercept for social strata, a constructed variable with subgroups based on an individual’s combination of age, race/ethnicity, insurance status, and RUCA category variables. P-values are based on type 3 sum of squares

Then, we examined differences in PND diagnosis and found significantly lower odds of PND diagnosis for all racial and ethnic groups when compared to White non-Hispanic women (Table 4B). Furthermore, women with no insurance had significantly lower odds of a PND diagnosis as compared to those with private insurance (OR: 0.62, CI: 0.48–0.81, *p* < 0.001), while women with public insurance showed 41% higher odds of PND diagnosis (OR: 1.35, CI: 1.24–1.61, *p* < 0.001). Women whose primary language was not English had 64% lower odds of a PND diagnosis as compared to primarily English-speaking women (OR: 0.36, CI: 0.30–0.44, *p* < 0.001). The strata with the 5 lowest prevalence rates for diagnosis (1.25–2.25%) were mostly Asian women with no insurance living in mostly urban areas. The strata with the highest diagnosis rates (23.3–25.6%) were all White non-Hispanic mothers on public insurance plans (Table 3B).

## Discussion and conclusions

This study contributes to the growing body of evidence documenting disparities in perinatal depression (PND) screening and diagnosis across racial, ethnic, and sociodemographic characteristics. Our findings reveal a paradox: while non-Hispanic Black, Hispanic, and Asian mothers were more likely to be screened for PND than their Non-Hispanic White counterparts, they were significantly less likely to receive a formal diagnosis. This pattern echoes prior research, which found that despite similar or higher rates of positive depression screens among Black and multiracial women, these groups were less likely to be diagnosed with depression during pregnancy (Sidebottom et al. 2021, 2023).

The underdiagnosis of depression among racial and ethnic minority women, particularly Black women, has been previously documented (Haight et al. 2024; Kozhimannil et al. 2011; Tabb et al. 2023). Taken from both quantitative and qualitative insights, Black and Hispanic women were less likely to receive a diagnosis or treatment for postpartum depressive symptoms, despite comparable or higher symptom prevalence (Haight et al. 2024; Hsieh et al. 2021; Kozhimannil et al. 2011). Our study also found that women with public insurance were more likely than those with private insurance to be diagnosed with PND, a finding that aligns with prior research suggesting that socioeconomic status influences both access to care and provider decision-making (Kozhimannil et al. 2011; Sidebottom et al. 2023). However, uninsured women were significantly less likely to be screened or diagnosed, highlighting a critical gap in care for this vulnerable population. This is consistent with other research noting that women on public insurance were more likely to have a positive depression screen or a diagnosis for depression, and that insurance status was a key determinant of postpartum depression care among low-income women (Kozhimannil et al. 2011; Sidebottom et al. 2023). Our study found that the intersection between race and socioeconomic status influenced the PND diagnosis, with Asian and Hispanic women with no insurance having the lowest probabilities of receiving a diagnosis.

Geographic disparities also emerged in our analysis. Women residing in rural areas were less likely to be screened for PND, a finding that mirrors national trends in mental health service access. The lower screening rates in rural settings may reflect limited provider availability, fewer integrated behavioral health services, and logistical barriers such as transportation. Using data from the Pregnancy Risk Assessment Monitoring System, our team previously found that the odds of PND were higher by 21% for women residing in rural compared to urban geographies. However, this association became tempered when we adjusted for maternal education, health insurance coverage, and WIC participation (Nidey et al. 2020). Our current study found that the intersection of geography, age, race, and insurance status influenced the probability of PND screening, with older non-Hispanic White women without insurance living in rural areas having the lowest likelihood of being screened for PND.

The discrepancy between screening and diagnosis rates, particularly among racial and ethnic minority women, raises important questions about the clinical interpretation of screening tools. While tools like the PHQ-9 and EPDS are widely validated, their sensitivity and specificity may vary across populations. Several studies note that cultural differences in symptom expression, language barriers, and provider bias may contribute to underdiagnosis, even when screening scores are elevated (Haight et al. 2024; Iturralde et al. 2021; Sidebottom et al. 2021, 2023). This suggests a need for more nuanced screening protocols that acknowledge intersectional identities and patient-centered communication. Community-based outreach and contextually specific interventions may help bridge the gap between screening and diagnosis for underserved populations. Despite national guidelines recommending universal screening for PND (Siu et al. 2016) our study and others demonstrate that implementation remains inconsistent and inequitable. Our findings support the use of intersectional analytic frameworks, such as MAIHDA, to uncover complex patterns of disparity. For example, the strata with the lowest predicted screening rates were composed of older, uninsured women in rural areas, while the highest screening rates were observed among younger, insured women in urban settings. These intersectional insights can inform more precise and equitable policy interventions.

This study benefits from its inclusion of a population from a single comprehensive health system, which enabled the identification of potential care gaps. The large, diverse sample size enhances the robustness of the findings and reflects real-world clinical settings. Additionally, using depression screening tools embedded within the EHR allows for more accurate tracking of screening practices. However, this study also has limitations. Notably, the reliance on EHR data may limit the ability to fully explore the underlying causes of observed inequities. Also, as this was a single health system within a Midwestern state, the findings may not be generalizable to other regions with different demographic profiles or patterns of prenatal and postpartum care. Conversely, as this study included data only from a single health system, future studies are warranted to determine results and trends from other settings administering depression screenings, such as women receiving care in other health systems, WIC and other community health offices or home visiting programs (Tabb et al. 2022; Tandon et al. 2011). Finally, this study is limited to PND specifically and does not address the myriad of co-occurring perinatal mood and anxiety disorders experienced by the perinatal population (Dossett et al. 2024).

In conclusion, our study highlights persistent and multifaceted disparities in PND screening and diagnosis. These disparities are not merely artifacts of individual behavior or provider oversight but reflect deeper structural inequities in the healthcare system. Addressing them will require coordinated efforts across healthcare systems to integrate mental health into all aspects of maternal care, such as routine and repeated screenings across the perinatal period (Dagher et al. 2021; Fedock and Alvarez 2018; Sidebottom et al. 2021; Yang et al. 2024). Additionally, to support more equitable distribution of mental health services, the healthcare system needs to develop support systems in underserved communities with targeted interventions. Lastly, the implementation of these mental health programs needs to be evaluated and scaled to achieve successful implementation (Haight et al. 2024; Sidebottom et al. 2021; Yang et al. 2024). Moving forward, a focus on equity, integration, and accountability is essential to improve outcomes for all families.

## Data Availability

Data used to create the dataset contains potentially sensitive information, and Indiana University and the Regenstrief Institute manage restrictions on the sharing of this information. Data can be requested through the Regenstrief Institute’s Data Services. Code can be requested from the Biostatistics Consulting Center at Indiana University (biostats@iu.edu).

## References

[CR1] Bates D, M. M, Bolker B, Walker S (2015) Fitting linear mixed-effects models using lme4. J Stat Softw 67(1):1–48

[CR2] Brown CC, Kuhn S, Stringfellow K, Moore JE, Ayers B (2023) Association between mental health conditions at the hospitalization for birth and postpartum hospital readmission. J Womens Health (Larchmt) 32(9):982–991. 10.1089/jwh.2022.0481 37327368 10.1089/jwh.2022.0481 PMC10517316

[CR3] Dagher R, Bruckheim H, Colpe L, Edwards E, White D (2021) Perinatal depression: challenges and Opportunities - PubMed. J Womens Health (Larchmt) 30(2). 10.1089/jwh.2020.8862 10.1089/jwh.2020.8862 PMC7891219 33156730

[CR4] Daw JR, MacCallum-Bridges CL, Admon LK (2025) Trends and disparities in maternal self-reported mental and physical health. JAMA Intern Med. 10.1001/jamainternmed.2025.1260 40423961 10.1001/jamainternmed.2025.1260 PMC12117492

[CR5] Dossett EC, Stuebe A, Dillion T, Tabb KM (2024) Perinatal mental health: the need for broader understanding and policies that meet the challenges. Health Aff (Millwood) 43(4):462–469. 10.1377/hlthaff.2023.01455 38560796 10.1377/hlthaff.2023.01455

[CR6] Evans CR, Leckie G, Subramanian SV, Bell A, Merlo J (2024) A tutorial for conducting intersectional multilevel analysis of individual heterogeneity and discriminatory accuracy (MAIHDA). SSM - Population Health 26:101664. 10.1016/j.ssmph.2024.101664 38690117 10.1016/j.ssmph.2024.101664 PMC11059336

[CR7] Fasial-Cury A, T. K, Ziebold C, Matijasevich A (2021) The impact of postpartum depression and bonding impairment on child development at 12 to 15 months after delivery. J Affect Disord Rep 4:100125. 10.1016/j.jadr.2021.100125

[CR8] Fedock G, Alvarez C (2018) Differences in screening and treatment for antepartum versus postpartum patients: are providers implementing the guidelines of care for perinatal Depression? - PubMed. J Womens Health (Larchmt) 27(9). 10.1089/jwh.2017.6765 10.1089/jwh.2017.6765 29757074

[CR9] Haight S, Daw J, Martin C, Sheffield-Abdullah K, Verbiest S, Pence B, Maselko J (2024) Racial and ethnic inequities in postpartum depressive Symptoms, Diagnosis, and care in 7 US Jurisdictions - PubMed. Health Aff 43(4). 10.1377/hlthaff.2023.01434 10.1377/hlthaff.2023.01434 PMC13034582 38560804

[CR10] Hsieh WJ, Sbrilli MD, Huang WD, Hoang TM, Meline B, Laurent HK, Tabb KM (2021) Patients’ perceptions of perinatal depression screening: a qualitative study. Health Aff (Millwood) 40(10):1612–1617. 10.1377/hlthaff.2021.00804 34606357 10.1377/hlthaff.2021.00804

[CR11] Iturralde E, Hsiao CA, Nkemere L, Kubo A, Sterling SA, Flanagan T, Avalos LA (2021) Engagement in perinatal depression treatment: a qualitative study of barriers across and within racial/ethnic groups. BMC Pregnancy Childbirth 21(1):512. 10.1186/s12884-021-03969-1 34271852 10.1186/s12884-021-03969-1 PMC8284181

[CR12] Kingston D, Tough S, Whitfield H (2012) Prenatal and postpartum maternal psychological distress and infant development: a systematic review. Child Psychiatry Hum Dev 43(5):683–714. 10.1007/s10578-012-0291-4 22407278 10.1007/s10578-012-0291-4

[CR13] Kozhimannil KB, Trinacty CM, Busch AB, Huskamp HA, Adams AS (2011) Racial and ethnic disparities in postpartum depression care among low-income women. Psychiatr Serv 62(6):619–625. 10.1176/ps.62.6.pss6206_0619 21632730 10.1176/appi.ps.62.6.619 PMC3733216

[CR14] Lusskin SI, Pundiak TM, Habib SM (2007) Perinatal depression: hiding in plain sight. Can J Psychiatry 52(8):479–488. 10.1177/070674370705200802 17955909 10.1177/070674370705200802

[CR15] Nidey N, Tabb KM, Carter KD, Bao W, Strathearn L, Rohlman DS, Wehby G, Ryckman K (2020) Rurality and risk of perinatal depression among women in the united States. J Rural Health 36(1):9–16. 10.1111/jrh.12401 31602705 10.1111/jrh.12401

[CR16] Palmer EOC, Ker S, Renteria ME, Carmody T, Rush AJ (2024) Psychometric evaluation and linking of the PHQ-9, QIDS-C, and VQIDS-C in a real-world population with major depressive disorder. Neuropsychiatr Dis Treat 20:671–687. 10.2147/NDT.S444223 38559772 10.2147/NDT.S444223 PMC10981376

[CR17] Sahebi A, Kheiry M, Abdi K, Qomi M, Golitaleb M (2024) Postpartum depression during the COVID-19 pandemic: an umbrella review and meta-analyses. Front Psychiatry 15:1393737. 10.3389/fpsyt.2024.1393737 39050914 10.3389/fpsyt.2024.1393737 PMC11266160

[CR18] Sidebottom A, Vacquier M, LaRusso E, Erickson D, Hardeman R (2021) Perinatal depression screening practices in a large health system: identifying current state and assessing opportunities to provide more equitable care - PubMed. Arch Women Ment Health 24(1). 10.1007/s00737-020-01035-x 10.1007/s00737-020-01035-x PMC7929950 32372299

[CR19] Sidebottom A, Vacquier M, LaRusso E, Schulte A, Nickel A (2023) Prenatal and postpartum depression diagnosis in a large health system: prevalence and disparities - PubMed. Ann Med 55(2). 10.1080/07853890.2023.2281507 10.1080/07853890.2023.2281507 PMC10836261 37963220

[CR20] Simonovich SD, Nidey NL, Gavin AR, Pineros-Leano M, Hsieh WJ, Sbrilli MD, Ables-Torres LA, Huang H, Ryckman K, Tabb KM (2021) Meta-analysis of antenatal depression and adverse birth outcomes in US populations, 2010-20. Health Aff (Millwood) 40(10):1560–1565. 10.1377/hlthaff.2021.00801 34606360 10.1377/hlthaff.2021.00801

[CR21] Siu AL, Force USPST, Bibbins-Domingo K, Grossman DC, Baumann LC, Davidson KW, Ebell M, Garcia FA, Gillman M, Herzstein J, Kemper AR, Krist AH, Kurth AE, Owens DK, Phillips WR, Phipps MG, Pignone MP (2016) Screening for depression in adults: US preventive services task force recommendation statement. JAMA 315(4):380–387. 10.1001/jama.2015.18392 26813211 10.1001/jama.2015.18392

[CR22] Sjoberg DD, Curry WK, Lavery M, Larmarange JA J (2021) Reproducible summary tables with the Gtsummary package. R J 13(1):570–580

[CR23] Stefana A, Cena L, Trainini A, Palumbo G, Gigantesco A, Mirabella F (2024) Screening for antenatal maternal depression: comparative performance of the Edinburgh postnatal depression scale and patient health Questionnaire-9. Ann Ist Super Sanita 60(1):55–63. 10.4415/ANN_24_01_08 38920259 10.4415/ANN_24_01_08

[CR24] Tabb KM, Simonovich SD, Wozniak JD, Barton JM, Hsieh WJ, Klement C, Ostrowski ME, Lakhani N, Meline BS, Huang H (2022) WIC staff views and perceptions on the relationship between food insecurity and perinatal depression. Healthcare. 10.3390/healthcare11010068 36611527 10.3390/healthcare11010068 PMC9819437

[CR25] Tabb KM, Beck DC, Tilea A, Bell S, Sugg GA, Vance A, Schroeder A, Admon L, Zivin K (2023) The relationship between diagnosed antenatal depression and anxiety and adverse birth outcomes between 2009 and 2020. Gen Hosp Psychiatry 85:239–242. 10.1016/j.genhosppsych.2023.07.003 37567852 10.1016/j.genhosppsych.2023.07.003 PMC10874620

[CR26] Tandon SD, Perry DF, Mendelson T, Kemp K, Leis JA (2011) Preventing perinatal depression in low-income home visiting clients: a randomized controlled trial. J Consult Clin Psychol 79(5):707–712. 10.1037/a0024895 21806298 10.1037/a0024895

[CR27] Yang Y, Wang T, Wang D, Liu M, Lun S, Ma S, Yin J (2024) Gaps between current practice in perinatal depression screening and guideline recommendations: a systematic review - PubMed. Gen Hosp Psychiatry 89. 10.1016/j.genhosppsych.2024.04.011 10.1016/j.genhosppsych.2024.04.011 38733723

[CR28] Yin X, Zhang F, Shi Y (2023) Prevalence and factors associated with hyperphosphatemia in continuous ambulatory peritoneal dialysis patients: a cross-sectional study. Front Med (Lausanne) 10:1142013. 10.3389/fmed.2023.1142013 37122336 10.3389/fmed.2023.1142013 PMC10140417

